# A research programme to evaluate DBT-PTSD, a modular treatment approach for Complex PTSD after childhood abuse

**DOI:** 10.1186/s40479-019-0099-y

**Published:** 2019-03-05

**Authors:** Martin Bohus, Christian Schmahl, Thomas Fydrich, Regina Steil, Meike Müller-Engelmann, Julia Herzog, Petra Ludäscher, Nikolaus Kleindienst, Kathlen Priebe

**Affiliations:** 1 Institute of Psychiatric and Psychosomatic Psychotherapy, Central Institute of Mental Health, Medical Faculty Mannheim / Heidelberg University, J5, 68159 Mannheim, Germany; 2 McLean Hospital Harvard Medical School, Boston, MA USA; 3 Department of Psychosomatic Medicine and Psychotherapy, Central Institute of Mental Health, Medical Faculty Mannheim / Heidelberg University, Heidelberg, Germany; 4 Institute of Psychology, Faculty of Life Sciences, Humboldt University, Berlin, Germany; 5 Department of Clinical Psychology and Psychotherapy, Institute of Psychology, Goethe University, Frankfurt am Main, Germany

**Keywords:** Childhood abuse, Complex posttraumatic stress disorder, Dialectical behavioural therapy, Randomised controlled trial

## Abstract

**Background:**

Posttraumatic stress disorder (PTSD) after childhood abuse (CA) is often related to severe co-occurring psychopathology, such as symptoms of borderline personality disorder (BPD). The ICD-11 has included Complex PTSD as a new diagnosis, which is defined by PTSD symptoms plus disturbances in emotion regulation, self-concept, and interpersonal relationships. Unfortunately, the empirical database on psychosocial treatments for survivors of CA is quite limited. Furthermore, the few existing studies often have either excluded subjects with self-harm behaviour and suicidal ideation — which is common behaviour in subjects suffering from Complex PTSD. Thus, researchers are still trying to identify efficacious treatment programmes for this group of patients.

We have designed DBT-PTSD to meet the specific needs of patients with Complex PTSD. The treatment programme is based on the rules and principles of dialectical behavioural therapy (DBT), and adds interventions derived from cognitive behavioural therapy, acceptance and commitment therapy and compassion-focused therapy. DBT-PTSD can be provided as a comprehensive residential programme or as an outpatient programme. The effects of the residential programme were evaluated in a randomised controlled trial. Data revealed significant reduction of posttraumatic symptoms, with large between-group effect sizes when compared to a treatment-as-usual wait list condition (Cohen’s d = 1.5).

The first aim of this project on hand is to evaluate the efficacy of the outpatient DBT-PTSD programme. The second aim is to identify the major therapeutic variables mediating treatment efficacy. The third aim is to study neural mechanisms and treatment sensitivity of two frequent sequelae of PTSD after CA: intrusions and dissociation.

**Methods:**

To address these questions, we include female patients who experienced CA and who fulfil DSM-5 criteria for PTSD plus borderline features, including criteria for severe emotion dysregulation. The study is funded by the German Federal Ministry of Education and Research, and started in 2014. Participants are randomised to outpatient psychotherapy with either DBT-PTSD or Cognitive Processing Therapy. Formal power analysis revealed a minimum of 180 patients to be recruited. The primary outcome is the change on the Clinician-Administered PTSD Scale for DSM-5.

**Discussion:**

The expected results will be a major step forward in establishing empirically supported psychological treatments for survivors of CA suffering from Complex PTSD.

**Trial registration:**

German Clinical Trials Register: registration number DRKS00005578, date of registration 19 December 2013.

## Background

### Sequelae of child abuse

Childhood abuse (CA) is a serious and pervasive problem worldwide, with childhood sexual abuse reported by 18% of women and 8% of men [1], and childhood physical abuse by 22 and 25%, respectively [2]. Adult survivors of CA often live with significant consequences, including psychiatric disorders. The World Mental Health Survey including representative surveys in 21 countries found that childhood adversities account for 30% of all mental disorders across countries [3]. Cohort studies [4–8] and epidemiological studies [3, 9, 10] revealed the highest odds ratios for affective disorders, substance related disorders, borderline personality disorder (BPD) and posttraumatic stress disorder (PTSD). The latter is often associated with emotion dysregulation, dissociative symptoms, aversive self-concepts, and interpersonal difficulties. This results in a relatively high comorbidity: Between 30 and 60% of patients with BPD suffer from co-occuring PTSD and between 17 and 30% of patients with PTSD meet the criteria for BPD [11–15]. These co-occuring symptoms have been described under the terms Complex PTSD (cPTSD) and Disorders of Extreme Stress Not Otherwise Specified (DESNOS). Both the DSM-5 [16] and the ICD-11 [17] have taken the symptoms of cPTSD into account. The DSM-5 added symptoms to the PTSD diagnosis that have frequently been viewed as symptoms of cPTSD, such as distorted beliefs about self and others, dissociation, and reckless behaviour. The ICD-11 [17] includes a distinct cPTSD diagnosis that comprises the three main symptom clusters of PTSD along with enduring disturbances in the domains of affect, self, and interpersonal relationships. Several studies support the ICD-11 distinction between PTSD and cPTSD, and have found that a history of CA is strongly associated with the latter [18, 19]. 

### Economic costs of PTSD and co-occurring BPD

Apart from the individual suffering, economic costs for treatment and functional impairment (e.g., unemployment) are a heavy burden for the health care system. Data from our group on economic aspects of patients with PTSD and severe emotion dysregulation like BPD, in Germany indicate average direct and indirect costs of about €28.000 per patient (2/3 direct treatment costs) for a period of 1 year, and further indicate that within the German health system, co-morbidity as well as severity of PTSD is related to higher direct costs for healthcare [20, 21].

### Psychosocial treatments

Cognitive-behavioural therapies have been shown to be efficacious in treating adults with PTSD in general [22, 23]. The recently revised Clinical Practice Guideline for the Management of PTSD [24] strongly recommends the use of individual, trauma-focused psychotherapies that have a primary component of exposure and/or cognitive interventions. However, there has been little study of the efficacy of these therapies for PTSD related to CA in general and specifically in patients with co-occuring BPD-symptoms. Meta-analyses specifically studying psychological treatment effects in patients with PTSD related to CA yielded smaller effect sizes (medium effect size d = 0.7; [25]) and lower recovery rates in CA-related PTSD [26] than compared to those studies, that included all types of PTSD. Furthermore, studies on CA-related PTSD often excluded patients with substance abuse, dissociative disorders, BPD and suicidality [26].

There is a mixed data base on the impact of CA on PTSD treatment outcome. A randomised controlled trial (RCT) of eye movement desensitization and reprocessing (EMDR) found that PTSD related to a childhood trauma compared to PTSD related to an adulthood trauma was associated with less improvement and a lower remission rate [27]. Another study, which compared prolonged exposure (PE) with stress inoculation training, also found that patients with a childhood trauma showed less improvement [28]. However, several other studies found no evidence supporting a negative impact of CA on the treatment outcome after PE, EMDR and cognitive processing therapy (CPT) [29–33]. A most recent metaregression-analysis included 51 RCTs and suggested that childhood trauma was associated with a poorer response to psychological therapy (Karatzias, Murphy, Cloitre, Bisson, Roberts, Shevlin: Psychological interventions for ICD-11 complex PTSD symptoms: systematic review and meta-analysis, submitted).

There is also a mixed data base regarding the impact of a co-occuring BPD. Five studies documented no significant effects of co-morbid BPD on treatment outcome [34–38]. Notably, three studies of these excluded patients with current self-injurious behaviour [34–36]. One study, which compared individual cognitive-behavioural therapy to individual present-centered therapy for female survivors of childhood sexual abuse (CSA) found that all patients with a co-occurring BPD diagnosis dropped out of cognitive-behavioural therapy [39].

Thus, researchers are still trying to identify treatment models for adult survivors of CA with concurrent severe emotional dyscontrol or severe dysfunctional behaviours. Currently, the American Psychological Association [24] and the US Department of Veterans Affairs and Department of Defense [40] considers four psychological treatments for PTSD to have strong research support. Among these empirically evaluated treatments, one of the currently most promising approaches is CPT [41, 42]. CPT was originally developed as a group treatment for rape victims. Originally it comprised two components: cognitive interventions and written trauma accounts [43]. Chard [44] adapted CPT for victims of CSA by combining individual and group treatment. In an RCT comparing 17 weeks of this treatment to a wait-list control, the treatment was found to be highly efficacious [44]. However, this study did not report data for patients with BPD. Resick et al. [45] conducted an RCT to separate out the efficacy of the two components of CPT, and found that a treatment consisting of only the cognitive interventions was as successful in treating PTSD as was the combination of cognitive and exposure interventions, with lower dropout rates. Accordingly, the written trauma accounts constitute no standard intervention of CPT anymore [42].

However, since personality disorders were not assessed in the RCT by Resick et al. [45], and since only 38% of the participants defined CSA as their worst traumatic event it remains unclear whether these treatment results can be generalised to patients with cPTSD related to CA. Moreover, the data revealed only small effects on typical problems of patients with cPTSD, such as difficulties in anger control and re-victimisation [46].

The International Society of Traumatic Stress Studies (ISTSS) recommends in its guidelines for the treatment of cPTSD the use of phase-based, modular treatments including modules to improve emotion regulation and traumatic memory processing [47]. One of the empirically best supported treatment for emotion dysregulation is dialectical behavioural therapy (DBT). However, in the absence of a specific protocol for treating co-occurring PTSD, only a minority of BPD clients with co-occurring PTSD who underwent 1 year of DBT treatment achieved full remission from PTSD [48, 49]. Accordingly, several treatments combining DBT and trauma-focused methods have been developed. Harned et al. [49, 50] added a PE protocol after successful stage I standard DBT. In a first open-trial study by Harned et al. [50], 13 BPD patients with PTSD received a trauma-focused exposure-based treatment in addition to ongoing standard outpatient DBT once they had achieved control over so-called stage I treatment targets such as self-injurious behaviour. Intent-to-treat analyses revealed significant improvement in posttraumatic symptoms and in most secondary outcomes, with medium to large pre-post effect sizes. In a second pilot RCT, Harned et al. [49] compared standard DBT (*n* = 9) with DBT + PE (*n* = 17). Eight of the 17 patients randomised to the DBT-PE arm started the PE protocol and only six patients (35%) completed the treatment. The sample size was too small for a sound interpretation of differential treatment effects. Cloitre et al. [51] reported the benefits of DBT-derived skills training as a precursor to PE as compared to supportive counselling as a precursor to PE in adults with PTSD after CA. The study yielded first evidence that a phase-based treatment including emotion regulation training might be superior to PE. However, the lack of a PE alone condition in this study precludes drawing conclusions about the relative benefits of the phased-treatment approach over state of the art PTSD treatment. Despite the promising results of these modified (phase-based) treatments, no treatment has yet been directly compared with a first-line PTSD treatment such as CPT.

### DBT-PTSD

As outlined above, none of established treatments met the requirements for a sufficient therapeutic approach for a population suffering from PTSD and co-occuring severe problems with emotion regulation, self-concept and social interaction. Accordingly, we developed DBT-PTSD to specifically address the needs of this group of patients. DBT-PTSD was designed to include severely sick patients, suffering from chronic CA-related cPTSD along with severe problems in emotion regulation, ongoing self-harm behaviour, suicidal ideations and dissociative symptoms, negative self-concepts with high levels of guilt, shame, self-contempt and interpersonal problems. DBT-PTSD has been developed as a disorder-specific multi-modular treatment concept with clear treatment algorithms. The backbone of DBT-PTSD, i.e. the principles, the rules, the majority of interventions and, in particular, the benevolent, challenging, “dialectical” attitude, are derived from DBT [52–54]. This concept, originally evaluated for chronic suicidal patients with BPD, includes clear structures and dynamic hierarchisation of treatment focuses. An additional significant element of DBT is the procurement of so-called “skills”. These are short and precise mental self-instructions and guidelines for action that aim to interrupt and modify automated, intrapsychic processing as well as behavioural patterns. The skills can be applied to manage extreme conditions of stress, tension and dissociation without problematic behaviour, to modulate maladaptive emotions and to modify automated cognitions. All of this plays a critical role in the successful treatment of cPTSD. Because trauma-specific interventions are not described in more detail in the standard DBT, we supplemented trauma-specific cognitive [55] and exposure-based techniques as described by Ehlers [55], and Foa et al. [56]. However, we had to consider, that within this group of patients, in-sensu exposure as described/applied in PE often goes with intense dissociative features, which hamper emotional learning [57–60] and therefore have negative impact on treatment outcome [61]. Accordingly, we modified the standard PE procedure [56] by adding anti-dissociative skills (skills-assisted exposure). Clinical experiences with patients with a history of CA have shown that early established cognitive-affective schemes often cannot be completely modified even by successful therapy. Therefore, it seems to be important that patients learn a better approach to these automated processes and implement a profound meta-cognitive and meta-emotional awareness, which enables them to process these automatic thoughts and emotional patterns from a more distanced perspective, learn to control the emotion driven action tendencies and replace them by functional behaviour. Acceptance and commitment therapy [62] provides valuable interventions here. Furthermore, this treatment consists of many helpful interventions for the recognition and implementation of values and therewith the improvement of the quality of life. Precisely because the self-concept is often characterized by trauma-related emotions such as shame or guilt, disgust and self-hate, many patients have significant difficulties dealing with themselves in a sympathetic and self-valuing manner – which is also frequently reflected in problems relating to interpersonal issues. In addition to the DBT-concept of teaching self-validation, in compassion-focused therapy [63], these difficulties are addressed through the training of a compassionate perspective towards oneself and other people. Here, compassion is defined as sensitivity towards one’s own suffering and that of other persons with a deep commitment to mitigate the suffering, and this thereby encompasses both an empathic, attentive and a purposeful, powerful component. All of these sources of DBT-PTSD are, in turn, anchored in the principles of mindfulness. Because many traumatized patients experience longer mindfulness meditations as unpleasant and encumbering at least at the beginning of the treatment, skills-based mindfulness is facilitated in DBT-PTSD. In this, the psychological active principles of mindfulness are portioned into individual skills suitable to a daily routine and shorter mindfulness exercises without relying on formal meditation as a necessary experience.

**Fig. 1:**
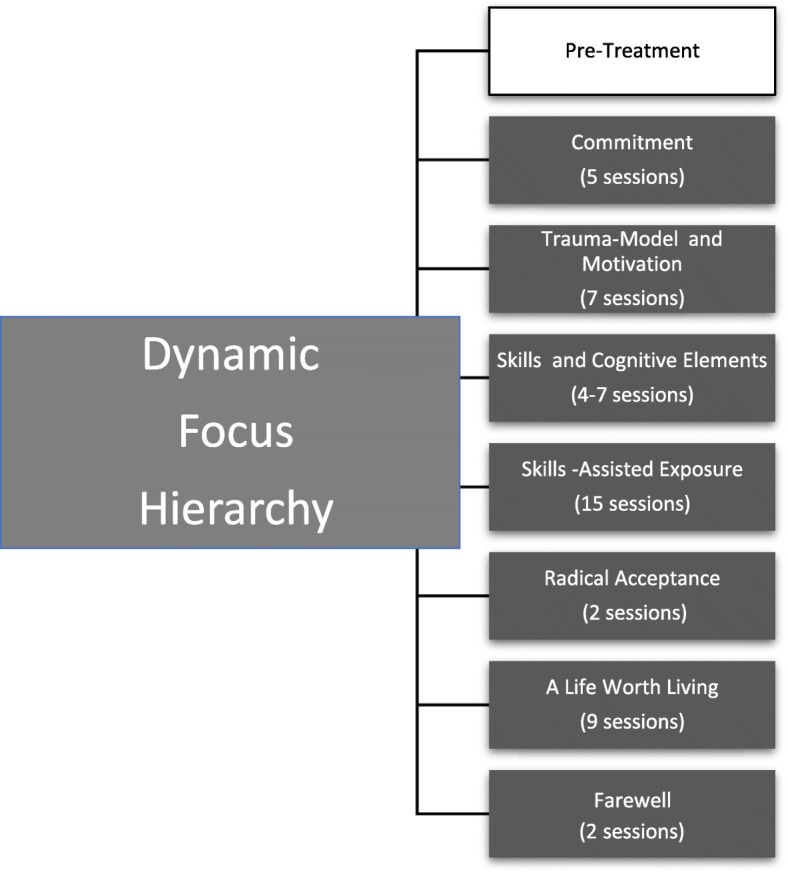
Treatment phases of DBT-PTSD with dynamic focus hierarchy

DBT-PTSD is divided into seven topical treatment phases (Fig. 1) which are spread out over 12 weeks in the inpatient setting and over 1 year with up to 45 individual therapy sessions in the outpatient setting. Every treatment phase includes mandatory and voluntary treatment modules. This modular approach allows to tailor the treatment to the diverse symptom constellations of individuals suffering from cPTSD. Manualized “if-then rules” help the therapists to decide which of the suitable modules is being used in the individual case.

Independent of the distinct treatment phases, all individual session agendas are determined by hierarchically ordered treatment targets, as follows: 1) reduce imminent life-threatening behaviours, 2) reduce behaviours interfering with treatment maintenance or progress. Skills training is provided during the individual therapy: Skills have been modified for the specific needs of patients with cPTSD, and include: 1) mindfulness and compassion skills, 2) distress tolerance skills, 3) emotion regulation skills, and 4) regaining a life worth living skills. Phone consultations with the individual therapist focus on crisis intervention. Consultation team meetings are aimed at maintaining treatment fidelity. These meetings are conducted by the therapists following guidelines in the treatment manual.

Diagnostics, indication of treatment, information on the treatment concept and the empirical database occur before the treatment begins (**Pre-Treatment Phase**). If the patient appears sufficiently motivated to start the treatment, a non-suicide contract will be concluded. In return, a crisis intervention telephone hotline is guaranteed to them.

In the **first phase (“Commitment”)**, a brief, structured interview records the active, dysfunctional behaviour pattern at the time. The therapist establishes a crisis and emergency plan. A short introduction to the skills concept also follows and, in this case in particular, an introduction to mindfulness. A special feature lies here in the development of a “compassionate-supporting self” (compassionate mindfulness). The patients develop this understanding by listening daily to imaginative self-instructions that have been recorded by their therapists. Specifically in this commitment phase, a rough orientation of time, manner and frequency of the traumatic experiences should be compiled, including the threats that the child was exposed to for reporting the trauma.

In **the second phase (“Trauma Model and Motivation”)**, the focus is on establishing a conclusive model of how PTSD develops, is preserved and can be treated. For this, the model of the “old path and the new path” with the trauma network and the mental and behaviour-related avoidance and escape strategies is generated. The patients should understand how strongly PTSD influences their lives and how trauma-related automatic thoughts and emotions prevent them from developing a meaningful life. The patients become acquainted with their typical avoidance and escape strategies and the related short- and long-term consequences. Moreover, they acquire a certain understanding for the mechanisms and effectiveness of exposure-based interventions (the brain must learn to differentiate between the past and the present). Based on this, therapist and patient develop operationalised, realistic and measurable treatment goals that are significant for the individual value system of the patient. Precisely because many patients with cPTSD have experienced severe disappointments from primary reference persons, we assume that these interpersonal experiences might repeat themselves in the therapeutic relationship in the context of transference processes and thereby hamper the cooperative work. In order to counteract this problem, we have taken up an idea from McCullough [64] and operate in advance an analysis of the experience with the most important formative significant others and their potential effect on the therapeutic relationship. The second treatment phase is completed with an analysis of potential disorder-maintaining conditions and individual fears regarding the therapy.

At the end of this second treatment phase, the patients and their therapists present the treatment plan together to the consultation team, mutually discuss the prospects of success as well as possible required support and obtain the permission to enter the third therapy phase – and, with it, permission to begin the preparation for the exposure phase.

In the **third phase (“Skills and Cognitive Elements”)**, the therapists analyse behaviour-related (e.g., self-harm) and emotional (e.g., guilt, shame, dissociation) escape strategies and convey the appropriate functional skills. The patients learn to recognise and rate the level of internal tension and learn to identify early signals of beginning dissociative states and to reduce them with strong sensory stimuli or physiological distraction (ice packs, ammonia, chili, juggling, eye-movement, and balancing). They also become familiar with the fundamental evolutionary significance of emotions such as guilt, shame, contempt and disgust and learn to recognise and modulate them.

The exposure-based processing of trauma-associated memories and emotions are at the core of the **fourth phase** (**“Skills-Assisted Exposure**”). To maintain the level of aversive arousal within a tolerable range and to prevent dissociative symptoms, the exposure occurs in accordance with the principle of the skills-assisted exposure. In doing so, the application of skills helps establishing a balance between activation of trauma-associated emotions and awareness of the present moment. The primary objective of this intervention is the exposure to the trauma-associated primary emotions such as powerlessness, disgust, anxiety and pain. In conformity with the theory of inhibitory learning, the intervention results in a reduction of these feelings which are not adequate for the present moment and in the correction of unrealistic fears in terms of a behaviour experiment (e.g., “I will go crazy if I allow this memory.”). Methodically, DBT-PTSD proceeds as follows: First the therapists and patients establish the so-called index trauma together. It has proved reasonable to select the incident here that is currently bound to the most frequent and most distressing intrusions and nightmares. During the later course of the phase, additional stressful memories can then be the focus. In the second step, the most critical fears and concerns are processed with respect to exposure: “I will go crazy.”, “I won’t survive it”. These fears are first substantiated and questioned in socratic dialogue. The actual exposure phase begins when the patients first write down the incident including thoughts and feelings that occurred during the event. This script is first read aloud in the therapy session. Then the repeated in-sensu exposure follows. In this, the therapists essentially strive to achieve a high emotional activation and to actively interrupt dissociative symptoms. Prototypically, the patient relates the traumatic experience during the exposure in the first person, in the present, with closed eyes. Intermittently, the exposure is interrupted by the therapists to create the sensory reference to reality: “What is the difference between then and now? How do you see this, how do you feel this?”. In the therapy session, the memory should be imagined at least two times. To ensure this, “hot spots” are selected and imagined. It is also helpful to ask at the end of the “hot spots” if the patient has reported anybody to this event and, if not, what prevented them from doing so. Often non-validating rejection by close caregivers is experienced as highly traumatic and should also be exposed. Between the therapy sessions, the patients listen to the audio recordings of the exposure every day at home. We have developed and evaluated an app that can easily impede dissociate symptoms during the exposure and can also monitor the processes of the emotions (decline in guilt, shame, disgust, etc.) (https://morpheus.deuschel-schueller.de). In most of the cases, a significant reduction in symptoms (decline in the frequency and distress from intrusions and flashbacks; revision of guilt and shame) sets in within 5 to 6 exposure sessions. Then the focus can be adjusted to other incidents in which the handling of generally requires less time and energy.

The **fifth phase (“Radical Acceptance”)** is linked to the exposure phase with exercises in acceptance and embracing what has been experienced. The majority of patients are still at odds with their past after the exposure phase and have marked difficulties in accepting this as unalterable and as an incident that happened. Patients often show concerns that acceptance could signify that the incidents were not so bad or that they have to forgive the perpetrators. Moreover, they show  emotional difficulties in parting from old illusions: “If I had only behaved appropriately, this would not have happened and I would have achieved an attentive, loving relationship with my father / mother”. This phase is also about ending the illusionary relationship with primary caregivers and making room for a mature, revised and realistic consideration. Embracing what has been experienced opens up space for grief which needs its own time.

In the **sixth phase (“A Life Worth Living”)**, the patients explore new areas of life or actively seek improvements to those factors which stand in the way of a meaningful life worth living. For patients with a history of CA, topics such as partnership and relationships, physical experience and sexuality as well as changes in the professional life are almost always significant. Precisely because not only the trauma-associated experience and behaviour but also the entire self-concept is to be accounted for in this therapy programme with very intense changes, the patients need structured support in this phase to develop a new living concept. Methodically, we rely on the model of the “old and new path” in this phase.

The **seventh and final phase (“Farewell**”) follows some predefined rules, since fear of abandonment is an intrinsic problem of patients with cPTSD. However, ending a therapeutic relationship after such an intense phase of cooperation naturally is a bit painful (for both, therapist and patient).

In summary, DBT-PTSD aims to help patients i) reduce their avoidance of trauma-associated primary emotions such as fear, disgust, pain, and powerlessness, ii) question non-justified trauma-related emotions such as guilt, shame, and self-contempt, and iii) radically accept trauma-related biographic facts. To successfully reduce avoidance of trauma-associated emotions, exposure-based techniques including control of escape strategies are required. Accordingly, patients learn to identify their typical cognitive, emotional, and behavioural escape strategies in response to trauma-related stimuli, and to use DBT skills to control these. The exposure protocol allows the patient to control the intensity of memory activation, and balances the vividness of trauma memories with the awareness of being in the (non-dangerous) present, by using skills during exposure sessions and exposure homework (skills-assisted exposure). Finally, the treatment focuses on relevant psychosocial aspects including work and partnership.

### The empirical base of DBT-PTSD

Following the usual process of evaluating a new treatment, as a first step, we carried out a non-controlled clinical pilot trial on residential DBT-PTSD treatment on 29 women who suffered from PTSD after CA and at least one other co-occurring diagnosis [65]. An effect size of Cohen's d = 1.22 on the Posttraumatic Diagnostic Scale (PDS; [66]) was found between baseline and the follow-up 6 weeks after the end of the 12-week programme. Effect sizes for secondary outcomes ranged from medium to large. The results suggested that DBT-PTSD is a very promising treatment for reducing severe PTSD after CA.

Next, we conducted a RCT in which women (*N* = 74) diagnosed with PTSD after CA plus at least one of the following additional diagnoses/symptoms: at least 4 DSM-IV criteria of BPD, current major depressive disorder, eating disorder, or alcohol and drug abuse were randomised to either the 12-week residential DBT-PTSD programme or a treatment-as-usual waiting list (TAU-WL) [67]. The two primary outcomes were scores on the Clinician-Administered PTSD Scale (CAPS; [68]) and on the PDS [66]. All measurements were done by blind raters. Only 6% of the DBT-PTSD group (2 out of 36) discontinued the treatment prematurely. Hierarchical linear models yielded statistically significant group*time effects, indicating a more pronounced improvement in the DBT-PTSD group compared to the TAU-WL, with large effect sizes. Between-group effect sizes were large for the CAPS (Hedges’s g = 1.57), PDS (g = 1.27), Global Assessment of Functioning (GAF, [69]; g = 1.31), and Beck Depression Inventory-II (BDI-II, [70]; g = 1.13). No patient in the DBT-PTSD group showed worsening of posttraumatic symptoms or exaggerated dysfunctional behaviour [67, 71]. Neither BPD symptom severity, the number of BPD criteria, nor self-harm behaviour had a significant influence on treatment outcome. Our findings provided clear evidence for the efficacy, high tolerability, and safety of the newly developed DBT-PTSD under residential conditions.

In preparation for the trial on hand, we have adapted the DBT-PTSD manual to outpatient conditions. DBT-PTSD now consists of a 1 year multi-component treatment providing up to 45 sessions à 50 min (37.5 h hours in total) of individual therapy (and telephone consultation as needed) plus up to 3 additional booster sessions during the following 3 months. These booster  sessions mainly focus on implementation of relevant treatment aspects in daily live. We have tested the manual in a pre-post pilot study that enrolled 20 patients with PTSD after CA plus at least 4 BPD criteria, and found similar results to those obtained under residential conditions (effect size CAPS: Cohen’s d = 1.5) [72].

Unlike current state-of-the-art treatments for PTSD, which typically consist of 12 to 16 sessions, we decided on a longer treatment duration and more treatment sessions as justified by the following:

- The included patients suffer not only from PTSD but also from severe emotional dysregulation and serious dysfunctional behavioural patterns including self-harm behaviour. Standard DBT programmes that do not focus on PTSD usually last at least 1 year [73–75];
- The successful residential programme consists of twice-weekly 45-min sessions of individual treatment (a total of 23 sessions over the 12 weeks) plus the following weekly group treatments: 90 min of skills training (11 sessions in total), 60 min of group intervention focusing on self-esteem (8 sessions in total), three 25 min mindfulness sessions (35 sessions in total), as well as 60 min of PTSD-specific psychoeducation (11 sessions in total) and three 90- min non-specific weekly group interventions (music therapy, art therapy).

In the trial on residential DBT-PTSD, we found an average reduction of PTSD symptoms as assessed by the CAPS from 88 at admission to 55 at discharge [67]. Despite this clinically significant reduction in the primary outcome, many of the patients needed further psychotherapy after discharge. Taken together, 45 sessions of outpatient treatment is a minimum, and is justified for scientific and ethical reasons. Furthermore, the German health care system in general covers the costs for up to 80 sessions of behaviour therapy. Designing a short-term therapy for this group of severely disturbed patients would be unrealistic and would not fit into the frame of the German health care system. To facilitate transfer into routine conditions, we further decided to provide all psychotherapeutic sessions in an individual format. This allows to disseminating these treatments to rural regions which have little access to psychotherapeutic supply.

### Cognitive processing therapy (CPT)

We chose CPT as an adequate active control condition. In close cooperation with P. Resick (Duke University, Durham, USA), the developer of this treatment, we have adapted the established protocol for CPT (cognitive only version) to a 1 year  outpatient individual therapy programme comprising up to 45 sessions plus up to 3 additional booster sessions during the following 3 months. Thus, dosage and frequency of both treatments are equal.

The protocol has been translated and has been approved by Dr. Resick, who serves as a cooperation partner and supervisor. We have further translated and adapted the rating for therapeutic adherence for CPT on the basis of the ones used by Resick et al. [45, 76].

To ensure treatment fidelity therapists receive 1.5 h per week team consultation with local supervisors. In addition, the local supervisors have monthly case-consultations with the treatment developer Dr. Resick.

Individual sessions follow a session-by-session protocol. In addition to building an effective therapeutic alliance, the first 4 sessions aim to obtain a detailed case history, patient’s specific problem behaviour, and emergency plans. The next 12 sessions contain the original 12 CPT core sessions, starting with psychoeducation about PTSD and the treatment rationale. After the patient has written an impact statement as to why the trauma has happened and how it affected her beliefs, cognitive restructuring is applied with regard to guilt and denial. Then, worksheets are introduced step by step which are intended to support the patient in identifying and changing other dysfunctional trauma-related beliefs (the so-called stuck-points) regarding safety, trust, control and power, self-esteem, and intimacy. From session 17 onwards, the content of the sessions is derived from the patient’s individual stuck point log. After working on the index trauma, and writing a second impact statement, other traumatic incidents can be the treatment focus. Towards the end of the treatment, other goals of the patient (positive activities, social relationships, vocational training or work issues) can be addressed using the already established cognitive techniques.

## Design

### Work plan

The three goals of this collaborative research group are:

1. To evaluate the efficacy and effectiveness of a newly developed outpatient psychotherapy programme tailored specifically for patients suffering from cPTSD after childhood physical and sexual abuse (Main project)
2. To assess the role of treatment integrity (therapeutic adherence and competence) for treatment outcome of DBT-PTSD and CPT (Adjunct project I)
3. To study the impact of successful treatments on the neurophysiological underpinnings of dissociation and intrusions (Adjunct project II)

### Main project

#### Hypotheses

1. Improvement of PTSD symptoms will be superior in DBT-PTSD as compared to CPT.
2. The superiority of DBT-PTSD over CPT is related to the severity of BPD symptoms at baseline.

#### Additional analyses

The collected data will enable us to additionally test potential moderator variables for both, general and differentiated treatment response: a) Client variables including severity of CA, age at the onset and duration of CA, pre-treatment severity of PTSD and dissociation, co-occurring depressive disorder, current age, and educational level; b) Therapist variables including length of experience and gender.

#### Health care costs

As cPTSD is related to very high health care costs, we will also address this aspect. Direct and indirect costs will be compared across treatments and will be further compared with costs related to other epidemiologically relevant mental disorders. The reference time frames will be 1 year before the start of the treatments, at the end of treatments, and at 1 year follow-up. State of the art methodology will be applied (e.g., [77–80]), including a questionnaire and a structured interview to assess health care costs.

#### Inclusion criteria

We include female subjects between the ages of 18 and 65 who have a primary diagnosis of DSM-5–defined PTSD related to childhood sexual abuse or childhood physical violence before the age of 18. In addition, the patients have to meet at least 3 DSM-5 criteria for BPD (including criterion 6, affective instability). Furthermore, the patients have to be available for 1 year of outpatient treatment with no scheduled absence of more than 4 weeks, have to understand the implications related to the participation in a clinical trial, and have to give their written informed consent prior to randomisation.

#### Exclusion criteria

Exclusion Criteria are a lifetime diagnosis of schizophrenia or bipolar I disorder, mental retardation, severe psychopathology requiring immediate treatment in a different setting (such as acute alcohol withdrawal syndrome, or BMI < 16.5), current substance dependence without abstinence within the last 2 month, life-threatening suicide attempts as assessed by the Severe Behaviour Dyscontrol Interview (SBDI, [81]) within the last 2 months, medical conditions contradicting exposure protocol (e.g., severe cardiovascular disorder), pregnancy, currently severe instable life situations (e.g., homelessness, or ongoing victimisation by the perpetrator), or treatment with CPT or DBT-PTSD within the last year. Patients with ongoing self-harm or high-risk behaviours are accepted in the study.

#### Randomisation and blinding procedures

Before the start of the study, patients who meet the eligibility criteria are randomised in 1:1 ratio to either DBT-PTSD or CPT. Concealed assignment to the treatment groups is assured by using an external, web-based randomisation service (http://randomizer.at, University of Graz, Austria). All persons involved in the diagnostics and ratings are blinded with respect to the group assignment.

#### Crisis management

Both interventions develop safety plans at treatment start, and employ active crisis intervention when needed. Similar to standards of care, therapists provide on-call after-hours service via area crisis services. Patients in both groups have access to emergency services when viewed as necessary by therapists in the respective conditions. Inpatient services are readily available across conditions and across sites.

#### Hospitalisation policy

Because of the nature of the population, some patients may require treatment in a psychiatric hospital, either at their own request or due to the concerns of care providers (usually due to high acute suicide risk). No outpatient therapist will serve in a responsible position for any patient (e.g., attending) during inpatient treatment. Study treatment will be terminated when patients are hospitalised for more than 2 weeks.

#### Psychotropic medication protocol

There is no established pharmacological treatment protocol for PTSD after CA. On the other hand, tapering patients off medication would restrict recruitment to a small number of patients with less severe pathology and would be at the cost of external validity. We decided to track week-by-week medications and medication changes in order to document any difference in medication management across conditions. The purpose of the study is not to evaluate the efficacy of a combination of psychotherapy and a psychopharmacologic algorithm, but rather to evaluate psychotherapy interventions under conditions similar to those encountered in the community.

#### Dropout policy

Besides patients prematurely terminating therapy, any patient who misses 6 consecutively scheduled weeks of individual therapy or stays longer than 2 weeks in a psychiatric hospital will be considered a drop-out from treatment. This rule was instituted because it can be very difficult to know exactly when an emotionally dysregulated patient has actually dropped out of therapy. Often, patients miss sessions because of mood swings, discouragement, or interfering day-to-day hassles, but they do not mean to drop out of treatment and they often change their minds within a short period of time. This drop-out rule will be explained to patients during the first session of individual therapy. It is conceivable that some patients achieve the treatment goals (full symptomatic remission) and want to terminate the treatment prior to 1 year of treatment. The study protocol foresees the possibility of an early remission: No longer fulfilling the diagnostic criteria of PTSD, as assessed by the Clinician-Administered PTSD Scale for DSM-5 (CAPS-5; [82, 83]), as well as the approval by the patient, the therapist and the supervisor. Patients who have achieved an early remission are not considered having dropped out of the study.

#### Protocols to prevent cross-contamination between DBT-PTSD and CPT conditions

To reduce contamination, CPT and DBT-PTSD are not provided by the same therapists. Treatment fidelity will be supported by regular team consultation, including video-based real-time supervision in both treatments. Protocol violations will be reported to the therapists immediately. Therapeutic training and experience of the therapists will be balanced across treatment groups.

#### Therapists, training, and treatment adherence

At each of the three sites, therapists have been either trained in DBT-PTSD or in CPT. DBT-PTSD therapists at each site have been trained by the treatment developers, CPT therapists at each site have been trained by Dr. Resick. Therapists were asked to videotape every session. A randomly selected 2 of the 45 sessions of each therapy are being evaluated with regard to treatment integrity. DBT-PTSD treatment adherence is evaluated by using the DBT-PTSD Adherence Rating Scale, which generates a global rating of DBT adherence as well as sub-scale strategy ratings for various DBT-PTSD strategy domains. CPT treatment adherence is evaluated using the CPT Adherence Ratings Scale, which is based on the adapted CPT treatment manual and orientated on the original CPT Therapists’ Adherence Protocol – Revised [84] and has been adapted to the present CPT manual by R. Steil’s workgroup [76].

#### Assessments

Assessments will be conducted by blind raters at intake and at months 3, 6, 9, 12, 15 (end of the treatment phase) and at 1 year follow up. In addition to these assessments, in DBT-PTSD weekly measurements will be completed in the form of a daily diary card, rating of suicidal ideation, non-suicidal self-injury, therapist notes, and pre- and post-session ratings for each individual session.

#### Screening measures

Structured Clinical Interview for DSM-IV Axis I Disorders (SCID-I; [85]); CAPS-5 [82, 83]; International Personality Disorder Examination – Borderline section (IPDE; [86]); SBDI [81]; Childhood Trauma Questionnaire (CTQ; [87]), Maltreatment and Abuse Chronology of Exposure Scale (MACE; [88]), Mehrfachwahl-Wortschatz-Intelligenztest (MWT; [89]).

#### Primary and secondary endpoints

The CAPS-5 [82, 83] will be used as the primary endpoint for testing hypotheses 1 and 2. In the first place testing will be based on the dimensional CAPS-scores assessed in the intent-to-treat sample. Secondary endpoints will include the Posttraumatic Stress Disorder Checklist for DSM-5 (PCL-5; [90]) to assess self-rated symptomatology of PTSD, the Dissociation Tension Scale (DSS-7, [91]), the short version of the Borderline Symptom List (BSL-23; [92]), the behavioural items of the Borderline Symptom List [93] including suicide attempts and non-suicidal self-injury, the BDI-II [70], and the GAF [69].

#### Power analyses

The sample size was determined from a formal power analysis. The study was designed to have sufficient statistical power (1-β ≥ 0.80) to detect a supposed medium-size superiority of DBT-PTSD over CPT for the time*treatment interaction. A no more than medium between-group effect-size (d = 0.5, which corresponds to an effect-size f(V) of 0.354 for the time*treatment contrast for DBT-PTSD vs. CPT within a repeated measures design) was assumed since CPT was supposed to be substantially more efficacious than the TAU-WL condition to which DBT-PTSD has been previously compared. Under these assumptions, data from 70 participants per group are required to achieve sufficient statistical power (1-β ≥ 0.80) for Hypothesis 1. For Hypothesis 2 (“the superiority of DBT-PTSD is related to the severity of BPD-symptoms at baseline”) the power analysis indicated that a sample-size of at least 90 per group is needed to achieve sufficient statistical power to detect a clinically meaningful incremental R^2^ of at least 0.1 within a regression model. Accordingly, the recruitment target was set at a minimum of 180 participants (90 per group) to be randomised. In order to recruit and treat a minimum of 180 patients within 2 years, the study is conducted at three large German centres: the Central Institute of Mental Health in Mannheim, the Institute of Psychology, Goethe University in Frankfurt, and the Institute of Psychology, Humboldt University in Berlin.

#### Statistical analysis

The CAPS-5 and the assessments pertaining to the secondary hypotheses are obtained at baseline (=T1); at month 3 (=T2); month 6 (=T3); month 9 (=T4); month 12 (=T5, end of high frequency phase); and month 15 (=T6, end low frequency phase = post assessment). Mixed linear models including these assessment points will be used as the primary analytic strategy to analyse and compare the changes in the two groups. Parameters will be estimated using restricted maximum likelihood estimates (REML) and without imposing predefined assumptions such as compound symmetry on the covariance matrix. Analyses are based on the intent-to-treat sample of patients who were randomised and fulfilled all inclusion criteria. To provide a more complete picture these primary analyses are complemented by analyses comprising those participants who completed the study according to protocol (ATP). To allow for a more comprehensive evaluation of the data, the results from the mixed linear models are supplemented by clinically meaningful indices including remission rates, response rates and effect sizes.

### Adjunct project I: Identifying key therapeutic components and competence as predictors of outcome in DBT-PTSD and CPT

#### Background

Treatment integrity, defined as the extent to which treatment is implemented as intended, is not only required in order to draw valid conclusions from clinical trials, but is also implicated as a key ingredient of treatment success [94, 95]. Treatment integrity includes 3 components: 1) treatment adherence, which refers to the degree of utilization of techniques as specified in a manual, 2) treatment differentiation, which implies that treatments in a study differ on relevant dimensions; and 3) therapeutic competence, which is defined as how well these techniques are delivered and adapted to the specific therapeutic context [96].

In controlled clinical trials, it is currently standard practice to control for treatment integrity by using adherence ratings, but there are considerably fewer studies assessing therapeutic competence and its relationship to outcome [95, 97, 98]. With respect to established treatments of PTSD, there are no published studies investigating adherence and competence as predictors of outcomes. One study investigated these variables in gestalt-derived treatment for survivors of CA, but only 54% of the sample met PTSD DSM-IV criteria [99]. Competence here was not significantly related to changes in interpersonal stress and emotional resolution; it should be noted, however, that ratings were delivered by non-expert raters. In contrast, previous trials with other disorders [100, 101] indicate that competence is a significant predictor of outcome and a better predictor than adherence. In both studies, improved methods to assess competence were used, which also covered specific treatment components.

Based on the finding that therapeutic alliance has been more consistently found to correlate with treatment outcome than competence correlates with treatment outcome [102, 103], it has often been argued that common factors may be more important than competences related to the specific treatment. However, findings from a meta-analysis [98] suggest that therapeutic alliance may be considered as a moderator of the relationship between competence and outcome, as indicated by larger effect sizes in studies that did not control for the influence of therapeutic alliance.

The present study aims at identifying therapeutic competencies that are predictive for effective treatment of PTSD, as well as its associations with adherence and alliance. In addition, the present study provides the possibility to compare two treatments, DBT-PTSD and CPT, with respect to general aspects of competence common to cognitive-behavioural treatments. Finally, the contribution of particular components of general and specific therapeutic competence to treatment response in both treatments, will be subject to secondary, exploratory analyses.

#### Hypotheses

- Therapists’ general therapeutic competencies will significantly predict treatment response (pre-post-changes of CAPS) in both treatments.
- Therapists’ DBT-PTSD–specific competencies will significantly predict treatment response at post-treatment in DBT-PTSD.
- Therapists’ CPT–specific competencies will significantly predict treatment response at post-treatment in CPT.

We expect that both general (hypothesis 1) and specific competencies (hypotheses 2a and 2b) will contribute significantly to the prediction of treatment response at post-treatment when controlling for pre-treatment severity of PTSD (pre-treatment scores of the CAPS) and BPD (pre-treatment scores of the BSL) as well as adherence and therapeutic alliance. To test hypotheses 2a and 2b, general competencies will also be controlled for.

#### Method

Assessments: Primary outcome measure will be the CAPS-5 [83]. Therapeutic competence will be assessed by two independent and extensively trained clinician raters who are blind to treatment outcome, using rating scales that have been specifically developed on the basis of the manuals applied in this trial (i.e. [104]).

Observer ratings of competence and adherence will be obtained from two randomly selected videotaped sessions per patient, covering two different treatment phases. The level of ratings averaged over the two phases will enter the path analyses. To assess general competencies, the Cognitive Therapy Scale [105] will be applied. This scale comprises 15 items (e.g., homework, guided discovery, efficient use of time, etc.). Items are rated on a scale ranging from 0 to 6, referring to the quality of implementation of interventions. The DBT-PTSD Competence Rating Scale [106] comprises 6 items referring to components specific for DBT-PTSD, such as appropriate implementation of skills. The CPT Competence Rating Scale [104] comprises 4 items reflecting competencies specific for CPT, such as identification of stuck points and optimal application of worksheets. For both treatment specific competence scales, items are rated on a scale from 0 to 6, and according to a detailed raters’ manual referring to the quality of implementation of interventions.

Adherence ratings will be assessed using the DBT-PTSD Adherence Rating Scale and the CPT Adherence Rating Scale, respectively, which both have been developed by our workgroup (i.e. [76]). To rate competence and adherence for one treatment session, on average 2 h are required.

All rating scales which have been developed to assess treatment integrity of DBT-PTSD and CPT as used in the present RCT have been evaluated for their psychometric properties e.g., with respect to inter-rater reliability (i.e., [76, 104]).

Therapeutic alliance will be assessed using the Helping Alliance Questionnaire [107]. This rating scale is an 11-item questionnaire that assess therapeutic alliance from both the patient’s and the therapist’s perspective. For example the patients’ version consists of 11 subtypes of patients’ helping alliances (e.g., the patient feels optimism and confidence that the therapist can help; the patient shares with the therapist similar conceptions of the aetiology of the problems). These components are rated on a 6-point Likert scale.

Data analyses: Path analyses will be carried out to determine the effects of the predictor variables listed above. For all hypotheses, differences in CAPS-5-scores at pre- vs. post (T6) will be specified as the criterion variable. For hypothesis 1, path analyses models with general competencies as predictor variables will be specified. For hypotheses 2a and 2b, path analyses models with the specific therapeutic competencies as predictor variables will be specified. For all hypotheses, path analyses models with pre-treatment severity of PTSD and BPD (pre-treatment scores in the CAPS-5, pre-treatment scores in the BSL) as well as therapeutic adherence and therapeutic alliance as additional predictor variables will be carried out. For hypotheses 2a and 2b, general competence will also be included into the path analysis. For hypothesis 1, a multi-sample path analysis with the grouping variable treatment condition (DBT-PTSD vs. CPT) will be performed. Path analyses allow us to specify correlations between predictor variables, and will be carried out using Mplus version 7 [108]. Mplus offers several advantages for data analysis, such as effective ways of missing value imputation (FIML) and dealing with multi-level (nested) data.

### Adjunct project II: Experimental validation of therapy response

#### Background

Besides psychometric measures, behavioural and neurobiological data can be used to validate treatment effects. Several studies have shown that functional (and in part also structural) alterations in PTSD are amenable to change by psychotherapy [109]. While intrusions are characterized by increased traumatic memory processing, dissociation is related to reduced memory processing up to amnesia [110]. Clinically, intrusions are accompanied by physiological hyperarousal, while dissociation is characterized by reduced arousal and, in extreme cases, a shut-down of sensory and motor processes. Dissociative responses have been shown to be stress-related [111] and reduced startle responses during dissociative states have been demonstrated [112]. The interaction of dissociation and learning processes has been investigated: Pavlovian conditioning was disturbed during dissociation in patients with BPD [57], and dissociation predicted poor outcome of standard DBT and of DBT-PTSD [61, 113]. Recently, a neurobiological model of PTSD has been proposed, differentiating processing of intrusive hyperarousal as opposed to dissociation with emotional overmodulation [114]. Intrusive responses are characterized by increased sympathetic activity (elevated heart rate and blood pressure), whereas dissociative responses are characterized by no change or a decrease in heart rate [115]. On the neural level, several studies have demonstrated amygdala hyperactivity together with medial prefrontal hypoactivity [116–118] to be associated with intrusive hyperarousal. On the other hand, dissociative responses are characterized by an increase in medial prefrontal and insular activity [115, 119, 120]. Amygdala activity was negatively correlated with dissociation levels during an Emotional Working Memory Task (EWMT; [121]). As intrusions and dissociation interfere with attention, the Stroop task has been widely used in PTSD. PTSD patients demonstrated increased interference to trauma-related material in the Emotional Stroop Task (EST; e.g., [122–124]). Imaging studies have demonstrated over-activation in the dorsal anterior cingulate cortex (dACC) as well as the insula in PTSD related to sexual abuse [125, 126]. Psychotherapy response was found to be related to a decrease of this over-activation in sexual abuse-related PTSD [127].

#### Hypotheses

- Improvement of PTSD symptoms from pre to the end of the high frequency phase of treatment (∆CAPS T1-T5) is correlated with the reduction of dorsal ACC and anterior insula activity (∆BOLD-signal T1-T5) during trauma-related words in the Emotional Stroop Task (EST).
- Improvement of PTSD symptoms from pre to the end of the high frequency phase of treatment (∆CAPS T1-T5) is correlated with reduction of amygdala activity and heart rate increase (∆BOLD-signal T1-T5) during negative pictures in the Emotional Working Memory Task (EWMT).
- Exploratively, we will investigate potential differences regarding neural activation patterns between DBT-PTSD responders and CPT responders. As DBT-PTSD includes skills-assisted exposure, we assume that DBT-PTSD will have a stronger effect on intrusions than will CPT. At the neural level, we would therefore expect a stronger reduction of the neural activation patterns of intrusions in DBT-PTSD responders as compared to CPT responders.

Power analyses for the hypotheses, i.e., that “Improvement of PTSD symptoms is correlated with the reduction of i) dorsal ACC activity, ii) anterior insula activity, iii) amygdala activity, iv) heart rate increase” are all tested at the Bonferroni-adjusted level of α_1_ = 0.0125. A large effect (r = 0.5) is assumed for the hypotheses. The assumption of a large effect is in line with the results on Emotional Stroop interference published by Thomaes et al. [127] who found large correlations ranging from 0.64 and 0.74 between improvements on the CAPS and decreased activation of several regions including the dorsal ACC and the anterior insula. Under these assumptions, the sub-sample recruited at the Mannheim and Frankfurt sites will be sufficient to achieve sufficient statistical power of 1-β = 0.86 for rejecting each of the hypotheses at the adjusted α-level of 0.0125 (two-tailed).

#### Method

Patients from both the DBT-PTSD and CPT arms who have been recruited at Mannheim and Frankfurt will be included in Adjunct project II, in which fMRI and laboratory measurements will be done before and after the high frequency phase of the treatment. During fMRI, the EST (20 words per valence; valence types: neutral, negative, trauma-related, colour words; each word presented in four colours) will be conducted first. Following the EST, the EWMT will be conducted, a working memory task with neutral and negative distractors. Activity of brain regions as assessed by the BOLD-responses as well as sympathetic (heart rate) and parasympathetic activity (heart rate variability) will be measured. Acute dissociation will be assessed with the Dissociation Tension Scale (DSS-4; [128]) intrusions will be assessed with the subscale “Intrusions” of the Impact of Event Scale- Revised (IES-R; [129]). Imaging data will be acquired using a 3 Tesla MRI Scanner (TRIO, Siemens Medical Systems, Erlangen, Germany). Neural activation patterns will be correlated with dissociation and intrusion scores within a multiple regression analysis using SPM 8 (http://www.fil.ion.ucl.ac.uk/spm/).

## Discussion

We have designed DBT-PTSD as the first treatment programme specifically designed for cPTSD related to childhood abuse. A first RCT revealed large between group effect sizes as compared to treatment-as-usual under residential conditions. The study on hand is aimed to compare the newly designed treatment with an established evidence based state of the art programme – CPT. In addition to treatment effectiveness and efficacy, this study will provide a large data set of including 200 patients with 6 assessment points plus follow up. This will open the opportunity to enlarge our knowledge about the complexity and interrelatedness of psychopathology, neurocognitive patterns and neuroimaging. Considering the fact, that cPTSD is a new ICD-11 diagnosis, there is not only a strong need to understand predictors, moderators and mediators of treatment response, but to create hypotheses of differential treatment response to exposure based or pure cognitive treatments.

## Abbreviations

ATP: According to Protocol
BDI: Beck Depression Inventory
BMI: Body Mass Index
BPD: Borderline Personality Disorder
BSL-23: Borderline Symptom List – short version
CA: Childhood Abuse
CAPS: Clinician-Administered PTSD Scale
CAPS-5: Clinician-Administered PTSD Scale for DSM-5
CPT: Cognitive Processing Therapy
cPTSD: Complex Posttraumatic Stress Disorder
CSA: Childhood Sexual Abuse
CTQ: Childhood Trauma Questionnaire
dACC: Dorsal Anterior Cingulate Cortex
DBT: Dialectical Behavioural Therapy
DBT-PE: Dialectical Behavioural Therapy plus Prolonged Exposure
DBT-PTSD: Dialectical Behavioural Therapy for Complex PTSD
DESNOS: Disorders of Extreme Stress Not Otherwise Specified
DSM-5: Diagnostic and Statistical Manual of Mental Disorders -5th revision
DSS-7: Dissociation Tension Scale
EMDR: Eye Movement Desensitization and Reprocessing
EST: Emotional Stroop Task
EWMT: Emotional Working Memory Task
GAF: Global Assessment of Functioning
ICD-11: International Statistical Classification of Diseases and Related Health Problems 10th Revision
IES-R: Impact of Event Scale - Revised
IPDE: International Personality Disorder Examination – Borderline section
MACE: Maltreatment and Abuse Chronology of Exposure Scale
MWT: Mehrfachwahl-Wortschatz-Intelligenztest
PCL-5: Posttraumatic Stress Disorder Checklist for DSM-5
PDS: Posttraumatic Diagnostic Scale
PE: Prolonged Exposure
PTSD: Posttraumatic Stress Disorder
RCT: Randomised Controlled Trial
REML: Restricted Maximum Likelihood Estimates
SBDI: Severe Behaviour Dyscontrol Interview
SCID-I: Structured Clinical Interview for DSM-IV Axis I Disorders

## References

[1] Stoltenborgh M, Van Ijzendoorn MH, Euser EM, Bakermans-Kranenburg MJ (2011). A global perspective on child sexual abuse: meta-analysis of prevalence around the world. Child Maltreat.

[2] Stoltenborgh M, Bakermans-Kranenburg MJ, van Ijzendoorn MH, Alink LR (2013). Cultural–geographical differences in the occurrence of child physical abuse? A meta-analysis of global prevalence. Int J Psychol.

[3] Kessler RC, McLaughlin KA, Green JG, Gruber MJ, Sampson NA, Zaslavsky AM (2010). Childhood adversities and adult psychopathology in the WHO world mental health surveys. Br J Psychiatry.

[4] Brown J, Cohen P, Johnson JG, Smailes EM (1999). Childhood abuse and neglect: specificity of effects on adolescent and young adult depression and suicidality. J Am Acad Child Adolesc Psychiatry.

[5] Cutajar MC, Mullen PE, Ogloff JR, Thomas SD, Wells DL, Spataro J (2010). Psychopathology in a large cohort of sexually abused children followed up to 43 years. Child Abuse Negl.

[6] Fergusson DM, McLeod GF, Horwood LJ (2013). Childhood sexual abuse and adult developmental outcomes: findings from a 30-year longitudinal study in New Zealand. Child Abuse Negl.

[7] Scott KM, Smith DR, Ellis PM (2010). Prospectively ascertained child maltreatment and its association with DSM-IV mental disorders in young adults. Arch Gen Psychiatry.

[8] Spataro J, Mullen PE, Burgess PM, Wells DL, Moss SA (2004). Impact of child sexual abuse on mental health: prospective study in males and females. Br J Psychiatry.

[9] Green JG, McLaughlin KA, Berglund PA, Gruber MJ, Sampson NA, Zaslavsky AM (2010). Childhood adversities and adult psychiatric disorders in the national comorbidity survey replication I: associations with first onset of DSM-IV disorders. Arch Gen Psychiatry.

[10] Pérez-Fuentes G, Olfson M, Villegas L, Morcillo C, Wang S, Blanco C (2013). Prevalence and correlates of child sexual abuse: a national study. Compr Psychiatry.

[11] Grant BF, Chou SP, Goldstein RB, Huang B, Stinson FS, Saha TD (2008). Prevalence, correlates, disability, and comorbidity of DSM-IV borderline personality disorder: results from the wave 2 national epidemiologic survey on alcohol and related conditions. J Clin Psychiatry..

[12] Lenzenweger MF, Lane MC, Loranger AW, Kessler RC (2007). DSM-IV personality disorders in the national comorbidity survey replication. Biol Psychiatry.

[13] McGlashan TH, Grilo CM, Skodol AE, Gunderson JG, Shea MT, Morey LC (2000). The collaborative longitudinal personality disorders study: baseline axis I/II and II/II diagnostic co-occurrence. Acta Psychiatr Scand.

[14] Pagura J, Stein MB, Bolton JM, Cox BJ, Grant B, Sareen J (2010). Comorbidity of borderline personality disorder and posttraumatic stress disorder in the U.S. population. J Psychiatr Res.

[15] Yen S, Shea MT, Battle CL, Johnson DM, Zlotnick C, Dolan-Sewell R (2002). Traumatic exposure and posttraumatic stress disorder in borderline, schizotypal, avoidant, and obsessive-compulsive personality disorders: findings from the collaborative longitudinal personality disorders study. J Nerv Ment Dis.

[16] American Psychiatric Association. Diagnostic and statistical manual of mental disorders (DSM-5®). American Psychiatric Pub. Arlington; 2013.

[17] World Health Organization. ICD-11. International Classification of Diseases 11^th^ revision. 2018. https://icd.who.int/. Accessed 21 Sept 2018.

[18] Cloitre M, Garvert DW, Brewin CR, Bryant RA, Maercker A (2013). Evidence for proposed ICD-11 PTSD and complex PTSD: a latent profile analysis. Eur J Psychotraumatol.

[19] Karatzias T, Shevlin M, Fyvie C, Hyland P, Efthymiadou E, Wilson D (2017). Evidence of distinct profiles of posttraumatic stress disorder (PTSD) and complex posttraumatic stress disorder (cPTSD) based on the new ICD-11 trauma questionnaire (ICD-TQ). J Affect Disord.

[20] Priebe K, Roth M, Krüger A, Glöckner-Fink K, Dyer A, Steil R (2017). Costs of mental health care in patients with posttraumatic stress disorder related to sexual abuse one year before and after inpatient DBT-PTSD. [article in German]. Psychiatr Prax.

[21] Wagner T, Fydrich T, Stiglmayr C, Marschall P, Salize HJ, Renneberg B (2014). Societal cost-of-illness in patients with borderline personality disorder one year before, during and after dialectical behavior therapy in routine outpatient care. Behav Res Ther.

[22] Cusack K, Jonas DE, Forneris CA, Wines C, Sonis J, Middleton JC (2016). Psychological treatments for adults with posttraumatic stress disorder: a systematic review and meta-analysis. Clin Psychol Rev.

[23] Watts BV, Schnurr PP, Mayo L, Young-Xu Y, Weeks WB, Friedman MJ (2013). Meta-analysis of the efficacy of treatments for posttraumatic stress disorder. J Clin Psychiatry..

[24] American Psychological Association. Clinical practice guideline for the treatment of posttraumatic stress disorder (PTSD) in adults. 2017. https://www.apa.org/ptsd-guideline/ptsd.pdf. Accessed 23 Sept 2018.

[25] Ehring T, Welboren R, Morina N, Wicherts JM, Freitag J, Emmelkamp PM (2014). Meta-analysis of psychological treatments for posttraumatic stress disorder in adult survivors of childhood abuse. Clin Psychol Rev.

[26] Dorrepaal E, Thomaes K, Hoogendoorn AW, Veltman DJ, Draijer N, van Balkom AJ (2014). Evidence-based treatment for adult women with child abuse-related complex PTSD: a quantitative review. Eur J Psychotraumatol.

[27] van der Kolk BA, Spinazzola J, Blaustein ME, Hopper JW, Hopper EK, Korn DL (2007). A randomized clinical trial of eye movement desensitization and reprocessing (EMDR), fluoxetine, and pill placebo in the treatment of posttraumatic stress disorder: treatment effects and long-term maintenance. J Clin Psychiatry.

[28] Hembree EA, Street GP, Riggs DS, Foa EB (2004). Do assault-related variables predict response to cognitive behavioral treatment for PTSD?. J Consult Clin Psychol.

[29] Resick PA, Nishith P, Griffin MG (2003). How well does cognitive-behavioral therapy treat symptoms of complex PTSD? An examination of child sexual abuse survivors within a clinical trial. CNS Spectr.

[30] Resick PA, Suvak MK, Wells SY (2014). The impact of childhood abuse among women with assault-related PTSD receiving short-term cognitive–behavioral therapy. J Trauma Stress.

[31] van Minnen A, Arntz A, Keijsers GPJ (2002). Prolonged exposure in patients with chronic PTSD: predictors of treatment outcome and dropout. Behav Res Ther.

[32] Wagenmans A, van Minnen A, Sleijpen M, de Jongh A (2018). The impact of childhood sexual abuse on the outcome of intensive trauma-focused treatment for PTSD. Eur J Psychotraumatol.

[33] Walter KH, Buckley A, Simpson JM, Chard KM (2014). Residential PTSD treatment for female veterans with military sexual trauma: does a history of childhood sexual abuse influence outcome?. J Interpers Violence.

[34] Clarke SB, Rizvi SL, Resick PA (2008). Borderline personality characteristics and treatment outcome in cognitive-behavioral treatments for PTSD in female rape victims. Behav Ther.

[35] Feeny NC, Zoellner LA, Foa EB (2002). Treatment outcome for chronic PTSD among female assault victims with borderline personality characteristics: a preliminary examination. J Personal Disord.

[36] Hembree EA, Cahill SP, Foa EB (2004). Impact of personality disorders on treatment outcome for female assault survivors with chronic posttraumatic stress disorder. J Personal Disord.

[37] Kredlow MA, Szuhany KL, Lo S, Xie H, Gottlieb JD, Rosenberg SD (2017). Cognitive behavioral therapy for posttraumatic stress disorder in individuals with severe mental illness and borderline personality disorder. Psychiatry Res.

[38] Holder N, Holliday R, Pai A, Surís A (2017). Role of Borderline personality disorder in the treatment of military sexual trauma-related posttraumatic stress disorder with cognitive processing therapy. Behav Med.

[39] McDonagh A, Friedman M, McHugo G, Ford J, Sengupta A, Mueser K (2005). Randomized trial of cognitive-behavioral therapy for chronic posttraumatic stress disorder in adult female survivors of childhood sexual abuse. J Consult Clin Psychol.

[40] US Department of Veteran Affairs. VA/DOD Clinical practice guideline for the management of posttraumatic stress disorder and acute stress disorder. 2017. https://www.healthquality.va.gov/guidelines/MH/ptsd/VADoDPTSDCPGFinal012418.pdf. Accessed 25 Sept 2018.

[41] Asmundson GJ, Thorisdottir AS, Roden-Foreman JW, Baird SO, Witcraft SM, Stein AT (2019). A meta-analytic review of cognitive processing therapy for adults with posttraumatic stress disorder. Cogn Behav Ther.

[42] Resick PA, Monson CM, Chard KM (2016). Cognitive processing therapy for PTSD: a comprehensive manual.

[43] Resick PA, Schnicke M (1993). Cognitive processing therapy for rape victims: a treatment manual.

[44] Chard KM (2005). An evaluation of cognitive processing therapy for the treatment of posttraumatic stress disorder related to childhood sexual abuse. J Consult Clin Psychol.

[45] Resick PA, Galovski TE, Uhlmansiek MO, Scher CD, Clum GA, Young-Xu Y (2008). A randomized clinical trial to dismantle components of cognitive processing therapy for posttraumatic stress disorder in female victims of interpersonal violence. J Consult Clin Psychol.

[46] Iverson KM, Resick PA, Suvak MK, Walling S, Taft CT (2001). Intimate partner violence exposure predicts PTSD treatment engagement and outcome in cognitive processing therapy. Behav Ther.

[47] Cloitre M, Courtois CA, Ford JD, Green BL, Alexander P, Briere J, et al. The ISTSS Expert Consensus Treatment Guidelines for Complex PTSD in Adults. 2012. http://www.istss.org/. Accessed 28 Sept 2018.

[48] Harned MS, Chapman AL, Dexter-Mazza ET, Murray A, Comtois KA, Linehan MM (2008). Treating co-occurring Axis I disorders in recurrently suicidal women with borderline personality disorder: a 2-year randomized trial of dialectical behavior therapy versus community treatment by experts. J Consult Clin Psychol.

[49] Harned MS, Korslund KE, Linehan MM (2014). A pilot randomized controlled trial of dialectical behavior therapy with and without the dialectical behavior therapy prolonged exposure protocol for suicidal and self-injuring women with borderline personality disorder and PTSD. Behav Res Ther.

[50] Harned MS, Korslund KE, Foa EB, Linehan MM (2012). Treating PTSD in suicidal and self-injuring women with borderline personality disorder: development and preliminary evaluation of a dialectical behavior therapy prolonged exposure protocol. Behav Res Ther.

[51] Cloitre M, Stovall-McClough KC, Nooner K, Zorbas P, Cherry S, Jackson CL (2010). (2010). Treatment for PTSD related to childhood abuse: a randomized controlled trial. Am J Psychiatry.

[52] Bohus M. Borderline-Störung. Fortschritte der Psychotherapie. Göttingen: Hogrefe-Verlag; 2002.

[53] Linehan MM. Cognitive-behavioral treatment of Borderline personality disorder. New York: Guilford Press; 1993.

[54] Linehan MM (2014). DBT skills training Manual.

[55] Ehlers A (1999). Posttraumatische Belastungsstörungen. Fortschritte der Psychotherapie.

[56] Foa EB, Hembree EA, Rothbaum BO (2007). Prolonged exposure therapy for PTSD: emotional processing of traumatic experiences.

[57] Ebner-Priemer UW, Mauchnik J, Kleindienst N, Schmahl C, Peper M, Rosenthal MZ (2009). Emotional learning during dissociative states in borderline personality disorder. J Psychiatry Neurosci.

[58] Cackowski S, Neubauer T, Kleindienst N (2016). The impact of posttraumatic stress disorder on the symptomatology of borderline personality disorder. Borderline Personal Disord Emot Dysregul.

[59] Krause-Utz A, Winter D, Schriner F, Chiu CD, Lis S, Spinhoven P (2018). Reduced amygdala reactivity and impaired working memory during dissociation in borderline personality disorder. Eur Arch Psychiatry Clin Neurosci.

[60] Vonderlin R, Kleindienst N, Alpers GW, Bohus M, Lyssenko L, Schmahl C (2018). Dissociation in victims of childhood abuse or neglect: a meta-analytic review. Psychol Med.

[61] Kleindienst N, Priebe K, Görg N, Dyer A, Steil R, Lyssenko L (2016). State dissociation moderates response to dialectical behavior therapy for posttraumatic stress disorder in women with and without borderline personality disorder. Eur J Psychotraumatol.

[62] Hayes CS, Strohsahl KD, Wilson KG (2016). Acceptance and commitment therapy: the process and practice of mindful change.

[63] Gilbert P (2010). Compassion focused therapy.

[64] McCullough JP (2014). CBASP as a distinctive treatment for persistent depressive disorder.

[65] Steil R, Dyer A, Priebe K, Kleindienst N, Bohus M (2011). Dialectical behavior therapy for posttraumatic stress disorder related to childhood sexual abuse: a pilot study of an intensive residential treatment program. J Trauma Stress.

[66] Griesel D, Wessa M, Flor H (2006). Psychometric qualities of the German version of the posttraumatic diagnostic scale (PTDS). Psychol Assess.

[67] Bohus M, Dyer AS, Priebe K, Krüger A, Kleindienst N, Schmahl C (2013). Dialectical behaviour therapy for post-traumatic stress disorder after childhood sexual abuse in patients with and without borderline personality disorder: a randomised controlled trial. Psychother Psychosom.

[68] Blake DD, Weathers FW, Nagy LM, Kaloupek DG, Gusman FD, Charney DS (1995). The development of a clinician-administered PTSD scale. J Trauma Stress.

[69] Endicott J, Spitzer RL, Fleiss JL, Cohen J (1976). The global assessment scale: a procedure for measuring overall severity of psychiatric disorders. Arch Gen Psychiatry.

[70] Beck A, Steer R, Brown G (1996). Beck depression inventory-II manual, ed 2.

[71] Krüger A, Kleindienst N, Priebe K, Dyer AS, Steil R, Schmahl C (2014). Non-suicidal self-injury during an exposure-based treatment in patients with posttraumatic stress disorder and borderline features. Behav Res Ther.

[72] Steil R, Dittmann C, Müller-Engelmann M, Dyer A, Maasch AM, Priebe K (2018). Dialectical behaviour therapy for posttraumatic stress disorder related to childhood sexual abuse: a pilot study in an outpatient treatment setting. Eur J Psychotraumatol.

[73] Linehan MM, Heard HL, Armstrong HE (1993). Naturalistic follow-up of a behavioral treatment for chronically parasuicidal borderline patients. Arch Gen Psychiatry.

[74] Linehan MM, Schmidt H, Dimeff LA, Craft JC, Kanter J, Comtois KA. Dialectical behavior therapy for patients with borderline personality disorder and drug-dependence. Am J Addict. 199l;8:279–92. 10.1080/105504999305686 10598211

[75] Linehan MM, Comtois KA, Murray AM, Brown MZ, Gallop RJ, Heard HL (2006). Two-year randomized controlled trial and follow-up of dialectical behavior therapy vs therapy by experts for suicidal behaviors and borderline personality disorder. Arch Gen Psychiatry.

[76] Dittmann C, Müller-Engelmann M, Resick PA, Gutermann J, Stangier U, Priebe K (2017). Adherence rating scale for cognitive processing therapy - cognitive only: analysis of psychometric properties. Behav Cogn Psychother.

[77] Krauth C, Hessel F, Hansmeier T, Wasem J, Seitz R, Schweikert B. Empirical standard costs for health economic evaluation in Germany - a proposal by the working group methods in health economic evaluation [German]. Gesundheitswesen. 2005;67:736–46. 10.1055/s-2005-858698 16235143

[78] Salize HJ, Kilian R (2010). Gesundheitsökonomie in der Psychiatrie. Konzepte, Methoden, Analysen.

[79] van Asselt AD, Dirksen CD, Arntz A, Severens JL (2007). The cost of borderline personality disorder: societal cost of illness in BPD-patients. Eur Psychiatry.

[80] van Asselt AD, Dirksen CD, Arntz A, Giesen-Bloo JH, van Dyck R, Spinhoven P (2008). Out-patient psychotherapy for borderline personality disorder: cost-effectiveness of schema-focused therapy v. Transference-focused psychotherapy. Br J Psychiatry.

[81] Bohus M, Borgmann E, Meinlschmidt G, Schneider S, Margraf J (2012). Borderline-Persönlichkeitsstörungen [Borderline personality disorder]. Lehrbuch der Verhaltenstherapie. Materialien für die Psychotherapie [Textbook of behavioural therapy. Materials for psychotherapy].

[82] Weathers FW, Blake DD, Schnurr PP, Kaloupek DG, Marx BP, Keane TM. The Clinician-Administered PTSD Scale for DSM-5 (CAPS-5). 2013. Interview available from the National Center for PTSD at www.ptsd.va.gov.

[83] Müller-Engelmann M, Schnyder U, Dittmann C, Priebe K, Bohus M, Thome J, et al. Psychometric properties and factor structure of the German version of the clinician-administered PTSD scale for DSM-5. Assessment. 2018;1073191118774840. 10.1177/1073191118774840 29766744

[84] Mcdonald A, Wiltsey-Stirman S, Wachen J, Resick P (2014). CPT therapist adherence and competence protocol – revised.

[85] First MB, Spitzer RL, Gibbon M, Williams JBW, Benjamin LS (1997). User’s guide for the structured clinical interview for DSM-IV Axis I disorders (SCID-I) – clinical version.

[86] Loranger AW, Sartorius N, Andreoli A, Berger P, Buchheim P, Channabasavanna SM (1994). The international personality disorder examination, IPDE. The WHO/ADAMHA international pilot study of personality disorders. Arch Gen Psychiatry.

[87] Bernstein DP, Fink LA (1998). CTQ: childhood trauma questionnaire: a retrospective self-report.

[88] Teicher MH, Parigger A (2015). The 'Maltreatment and abuse chronology of Exposure' (MACE) scale for the retrospective assessment of abuse and neglect during development. PLoS One.

[89] Lehrl S (1999). Mehrfachwahl-Wortschatz-Intelligenztest MWT-B.

[90] Blevins CA, Weathers FW, Davis MT, Witte TK, Domino JL (2015). The posttraumatic stress disorder checklist for DSM-5 (PCL-5): development and initial psychometric evaluation. J Trauma Stress.

[91] Stiglmayr C, Schimke P, Wagner T, Braakmann D, Schweiger U, Sipos V (2010). Development and psychometric characteristics of the dissociation tension scale. J Pers Assess.

[92] Bohus M, Kleindienst N, Limberger MF, Stieglitz RD, Domsalla M, Chapman AL (2009). The short version of the Borderline symptom list (BSL-23): development and initial data on psychometric properties. Psychopathology.

[93] Bohus M, Limberger MF, Frank U, Sender I, Gratwohl T, Stieglitz RD (2001). Development of the Borderline symptom list. [German]. Psychother Psychosom Med Psychol.

[94] Perepletchikova F, Treat TA, Kazdin AE (2007). Treatment integrity in psychotherapy research: analysis of the studies and examination of the associated factors. J Consult Clin Psychol.

[95] Weck F (2014). Psychotherapeutische Kompetenzen: Theorien, Erfassung, Förderung.

[96] Waltz J, Addis ME, Koerner K, Jacobson NS (1993). Testing the integrity of a psychotherapy protocol: assessment of adherence and competence. J Consult Clin Psychol.

[97] Perepletchikova F, Hilt LM, Chereji E, Kazdin AE (2009). Barriers to implementing treatment integrity procedures: survey of treatment outcome researchers. J Consult Clin Psychol.

[98] Webb CA, DeRubeis RJ, Barber JP (2010). Therapist adherence/competence and treatment outcome: a meta-analytic review. J Consult Clin Psychol.

[99] Paivio SC, Cramer KM (2004). Factor structure and reliability of the childhood trauma questionnaire in a Canadian undergraduate student sample. Child Abuse Negl.

[100] Barber JP, Crits-Christoph P, Luborsky L (1996). Effects of therapist adherence and competence on patient outcome in brief dynamic therapy. J Consult Clin Psychol.

[101] Ginzburg DM, Bohn C, Höfling V, Weck F, Clark DM, Stangier U (2012). Treatment specific competence predicts outcome in cognitive therapy for social anxiety disorder. Behav Res Ther.

[102] Keller SM, Zoellner LA, Feeny NC (2010). Understanding factors associated with early therapeutic alliance in PTSD treatment: adherence, childhood sexual abuse history, and social support. J Consult Clin Psychol.

[103] Martin DJ, Garske JP, Davis MK (2000). Relation of the therapeutic alliance with outcome and other variables: a meta-analytic review. J Consult Clin Psychol.

[104] Dittmann C, Müller-Engelmann M, Stangier U, Priebe K, Fydrich T, Görg N (2017). Disorder-and treatment-specific therapeutic competence scales for posttraumatic stress disorder intervention: development and psychometric properties. J Trauma Stress.

[105] Young J, Beck AT. Cognitive therapy scale: Rating manual. Unpubl Manuscr. 1980:36th.

[106] Steil R, Dittmann C, Müller-Engelmann M, Resick PA, Gutermann J, Priebe K, et al. Identifying key therapeutic components and competence as predictors of treatment outcome in DBT-PTSD and CPT. 2016; 5th German Health Research meeting on Behavioural disorders related to violence, neglect, maltreatment, and abuse in childhood and adolescence. Mannheim.

[107] Vallis TM, Shaw BF, Dobson KS. The cognitive therapy scale: psychometric properties. J Consult Clin Psychol. 1986;54:381-5. 10.1037//0022-006x.54.3.381 3722567

[108] Muthén LK, Muthén BO (2012). Mplus statistical modeling software: Release 7.0.

[109] Thomaes K, Dorrepaal E, Draijer N, Jansma EP, Veltman DJ, van Balkom AJ (2014). Can pharmacological and psychological treatment change brain structure and function in PTSD? A systematic review. J Psychiatr Res.

[110] Lanius RA, Brand B, Vermetten E, Frewen PA, Spiegel D (2012). The dissociative subtype of posttraumatic stress disorder: rationale, clinical and neurobiological evidence, and implications. Depress Anxiety.

[111] Stiglmayr CE, Ebner-Priemer UW, Bretz J, Behm R, Mohse M, Lammers CH (2008). Dissociative symptoms are positively related to stress in borderline personality disorder. Acta Psychiatr Scand.

[112] Ebner-Priemer UW, Badeck S, Beckmann C, Wagner A, Feige B, Weiss I (2005). Affective dysregulation and dissociative experience in female patients with borderline personality disorder: a startle response study. J Psychiatr Res.

[113] Kleindienst N, Limberger MF, Ebner-Priemer UW, Keibel-Mauchnik J, Dyer A, Berger M (2011). Dissociation predicts poor response to dialectial behavioral therapy in female patients with borderline personality disorder. J Personal Disord.

[114] Lanius RA, Vermetten E, Loewenstein RJ, Brand B, Schmahl C, Bremner JD (2010). Emotion modulation in PTSD: clinical and neurobiological evidence for a dissociative subtype. Am J Psychiatry.

[115] Lanius RA, Williamson PC, Bluhm RL, Densmore M, Boksman K, Neufeld RW (2005). Functional connectivity of dissociative responses in posttraumatic stress disorder: a functional magnetic resonance imaging investigation. Biol Psychiatry.

[116] Lanius RA, Williamson PC, Densmore M, Boksman K, Gupta MA, Neufeld RW (2001). Neural correlates of traumatic memories in posttraumatic stress disorder: a functional MRI investigation. Am J Psychiatry.

[117] Schmahl CG, Vermetten E, Elzinga BM, Douglas BJ (2003). Magnetic resonance imaging of hippocampal and amygdala volume in women with childhood abuse and borderline personality disorder. Psychiatry Res.

[118] Shin LM, Orr SP, Carson MA, Rauch SL, Macklin ML, Lasko NB (2004). Regional cerebral blood flow in the amygdala and medial prefrontal cortex during traumatic imagery in male and female Vietnam veterans with PTSD. Arch Gen Psychiatry.

[119] Lanius RA, Williamson PC, Boksman K, Densmore M, Gupta M, Neufeld RW (2002). Brain activation during script-driven imagery induced dissociative responses in PTSD: a functional magnetic resonance imaging investigation. Biol Psychiatry.

[120] Ludascher P, Valerius G, Stiglmayr C, Mauchnik J, Lanius RA, Bohus M (2010). Pain sensitivity and neural processing during dissociative states in patients with borderline personality disorder with and without comorbid posttraumatic stress disorder: a pilot study. J Psychiatry Neurosci.

[121] Krause-Utz A, Oei NY, Niedtfeld I, Bohus M, Spinhoven P, Schmahl C (2012). Influence of emotional distraction on working memory performance in borderline personality disorder. Psychol Med.

[122] McNally RJ, Kaspi SP, Riemann BC, Zeitlin SB (1990). Selective processing of threat cues in posttraumatic stress disorder. J Abnorm Psychol.

[123] Bryant RA, Harvey AG (1995). Processing threatening information in posttraumatic stress disorder. J Abnorm Psychol.

[124] Field NP, Classen C, Butler LD, Koopman C, Zarcone J, Spiegel D (2001). Revictimization and information processing in women survivors of childhood sexual abuse. J Anxiety Disord.

[125] Bremner JD, Vermetten E, Vythilingam M, Afzal N, Schmahl C, Elzinga B (2004). Neural correlates of the classic color and emotional stroop in women with abuse-related posttraumatic stress disorder. Biol Psychiatry.

[126] Herzog JI, Niedtfeld I, Rausch S, Thome J, Mueller-Engelmann M, Steil R, et al. Increased recruitment of cognitive control in the presence of traumatic stimuli in complex PTSD. Eur Arch Psychiatry Clin Neurosci. 2017;1–13. 10.1007/s00406-017-0822-x 28712089

[127] Thomaes K, Dorrepaal E, Draijer N, de Ruiter MB, Elzinga BM, van Balkom AJ (2012). Treatment effects on insular and anterior cingulate cortex activation during classic and emotional Stroop interference in child abuse-related complex post-traumatic stress disorder. Psychol Med.

[128] Stiglmayr C, Schmahl C, Bremner J, Bohus M, Ebner-Priemer U. Development and psychometric characteristics of the DSS-4 as a short instrument to assess state dissociative experience during neuropsychological experiments. Psychopathology. 2009;42:370–4. 10.1159/000236908 19752590

[129] Maercker A, Schützwohl M (1998). Erfassung von psychischen Belastungsfolgen: die impact of event Skala-revidierte version (IES-R) [assessment of post-traumatic stress reactions: the impact of event scale-revised (IES-R)]. Diagnostica.

